# A protocol to convert spatial polyline data to network formats and applications to world urban road networks

**DOI:** 10.1038/sdata.2016.46

**Published:** 2016-06-21

**Authors:** Alireza Karduni, Amirhassan Kermanshah, Sybil Derrible

**Affiliations:** 1 Complex and Sustainable Urban Networks (CSUN) Lab, Department of Civil and Materials Engineering, University of Illinois at Chicago, Chicago, Illinois 60607, USA; 2 School of Architecture, University of North Carolina at Charlotte, Charlotte, North Carolina 28223, USA

**Keywords:** Environmental sciences, Geography, Civil engineering

## Abstract

The study of geographical systems as graphs, and networks has gained significant momentum in the academic literature as these systems possess measurable and relevant network properties. Crowd-based sources of data such as OpenStreetMaps (OSM) have created a wealth of worldwide geographic information including on transportation systems (e.g., road networks). In this work, we offer a Geographic Information Systems (GIS) protocol to transfer polyline data into a workable network format in the form of; a node layer, an edge layer, and a list of nodes/edges with relevant geographic information (e.g., length). Moreover, we have developed an ArcGIS tool to perform this protocol on OSM data, which we have applied to 80 urban areas in the world and made the results freely available. The tool accounts for crossover roads such as ramps and bridges. A separate tool is also made available for planar data and can be applied to any line features in ArcGIS.

## Background & Summary

From rivers, roads and pipelines to electrical and telecommunication lines, many geographical systems are composed of collections of elements that are connected in space. Therefore, from studying and understanding human activities^1^ to studying the relationship between different urban areas^2^, conducting these studies through the lens of graphs and networks can shed light on many complex characteristics of the elements that are built upon them^3,4^. This approach is rooted in the origins of the field of Graph Theory developed in the 18th century by Euler and his Seven Bridges of Königsberg^5^, and it has been applied widely ever since^6–13^. The use of graphs is further reinforced in this era of ‘Big Data’, where countless sources of organizational or crowd-sourced data such as locational social media (Twitter geolocations, Facebook check-ins) and geographical data (road networks and public transportation systems through OpenStreetMaps (OSM) (http://www.openstreetmap.org), and businesses location data through Yelp and Google) have become available^14^.

Nevertheless, when focusing on geographical networks, and specifically on road systems, raw datasets often contain issues that make them unsuitable to conduct proper graph studies. Specifically, there are two main problems; first achieving a topologically correct dataset that represents the actual status of the street network as accurately as possible (topology problem), and second is developing a graph file format that is ready to be analyzed with available software and libraries (file format problem). In direct response to these problems, the main objective of this work is threefold; first to offer a formal protocol to convert Geographic Information Systems (GIS) data into a workable network format; second, to develop a tool to apply this protocol; and third to use the tool on a significant number of road systems and make the results available. For the purpose of this study we applied the tool to the road systems of some 80 most populated urban areas in the world (using available OSM data), and hope to supplement this database with the road networks of more cities in the future.

In the following section, we describe the existing tools and datasets that focus on network analysis of road networks followed by our methodology where, we offer a description of the protocol to effectively address the two problems mentioned earlier (topology and file format). Moreover, we offer a summary of the dataset that we have made publicly available. This is followed by a discussion of common issues that researchers might encounter when utilizing these datasets. Finally, we discuss how researchers can produce this data for any road network they might be interested in. The tool can be used with the commercial software package ArcGIS. The OSM tool takes into account roads that cross but do not intersect such as bridges and ramps when the information is present already in OSM. We have made a second version of the tool available that transforms any line feature that does not include elevation and intersection information (e.g., pipes, rivers, rails) into a spatial planar graph (e.g., a graph with no intersecting edges^15^). The latest version of the tools can be found on the Data & Tools page of the Complex and Sustainable Urban Networks (CSUN) Laboratory at http://csun.uic.edu/data.html#GISF2E (Accessed Jan 19 2016). Moreover, permanent versions of the tools along with the results for the 80 cities are permanently stored on Figshare (Data Citation 1, Data Citation 2).

There are several existing tools and software that enable researchers to conduct network and graph analyses. ArcGIS Network Analyst and QGIS Network Analysis Library are two popular toolsets, both of which create network datasets from road network files easily. However, the tools only allow users to conduct certain studies, such as shortest path calculations from a series of points to any other points, similar to origin destination matrices. Yet they do not provide a method to measure the whole system through a graph analysis and to calculate various graph metrics such as betweenness and closeness centralities^16^. Although ArcGIS Network Analyst allows some degrees of topology correction within the software’s ecosystem, there is no straightforward method to convert the network datasets to a workable graph format such as an edge list (i.e., list of edges/links) or an adjacency matrix (i.e., square matrix of all nodes, containing 0 or 1 s when two nodes are connected).

DepthMapX (https://varoudis.github.io/depthmapX/) which comes in the form of standalone software, as well as a plugin for QGIS, allows the user to calculate various network metrics for road systems, but only works for a certain type of graph analysis, Space Syntax, as developed by Bill Hillier^17^. DepthMapX works with axial maps, which are a specific type of spatial graphs^18^ as opposed to regular road maps, and takes the input data in many formats including AutoCAD (DWG format).

In contrast to the lack tools to convert a GIS line feature into a network, there are an abundance of libraries and software packages to conduct graph analyses, all varying in how much expertise is required to run them. Gephi^19^ is a graph analysis software with a simple and intuitive graphical user interface. NodeXL^20^ enables users to conduct graph analysis from Microsoft Excel. NetworkX^21^ and igraph^22^ are libraries for python that enable users to conduct graph analyses with minimal programming background. All of the mentioned libraries and software packages can input a series of standard graph file format such as an edge list and an adjacency matrix as described above.

In regards to the road system graph data, there are some datasets available, to name a few, the Stanford Large Network Dataset Collection (https://snap.stanford.edu/data/#road) consists of many ready graph datasets, which include road graph files for three American states. As well, the school of computing at the University of Utah also has a series of graph files for roads networks available as edge lists^23^. However, none of these datasets can be imported back in a GIS environment, and no information could be found on how topologically correct they are.

Our toolset and dataset bridge the gap between semi-enclosed ecosystems such as ArcGIS and QGIS, and graph analysis libraries such as Gephi and igraph. This is achieved by providing both shapefiles/feature classes and network edge lists that are connected to each other with unique identifiers. Our dataset enables GIS users to easily conduct graph analyses for road systems of the 80 most populated urban areas in the world, by providing accurate data that can be easily inputted into the various graph analysis libraries listed above. The results can then be imported into a GIS environment to conduct geographic analyses and visualizations^24^. The provided toolset will enable users to create topologically correct graph edge lists from OpenStreetap (OSM), and planar graph edge lists from any road network shapefile that lacks the required information. The toolset can in fact process any line features, from roads and rail systems, to water conduits, electrical systems and even rivers.

## Methods

**Figure 1:**
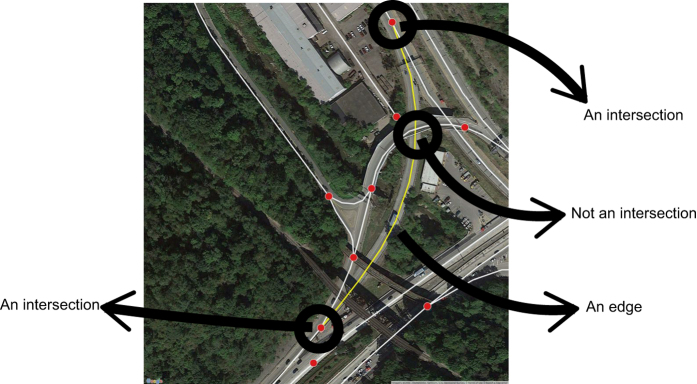
Superposing a generated graph on top of the real image of the area to define 3D aspects of the graph (i.e., define nodes and edges).

In a reverse engineering fashion we first note how we want our final results to be, which drives the entire procedure. Essentially, we need our network information to consist of a data set of nodes as intersections and a data set of edges/links as road segments that connect the nodes. Moreover, we need to calculate the length of edges in our dataset in a way that takes into account the curvature of the road segments. In addition we need to ensure, given the existence of required road information, any non-intersection such as highway over passes, bridges, or tunnels that overlap but that do not intersect are not counted as nodes in our graph system (Fig. 1).

To solve this problem, we establish the following five-step protocol:

1. Separate the data set into different road crossing categories based on OSM highways tags: (a) bridge and (b) tunnel
2. For each category:
  - Split the lines at their intersection
  - Create nodes at the start and end point of each split line
3. Merge the edges together
4. Merge the nodes together
5. Remove duplicate nodes

As mentioned before, our tool utilizes OSM data as the data source. The line data in OSM includes many crowd-sourced attribute tags, such as street type, name and so on. The key attribute information we have used to create our graph files are two specific OSM highway tags:

- bridge tag (0 or 1), which is defined as any road that crosses over another (See http://wiki.openstreetmap.org/wiki/Key:bridge);
- tunnel (0 or 1) which is defined as ‘any underground path for a road or similar’ (See http://wiki.openstreetmap.org/wiki/Key:tunnel). The required data format for our tool to perform is as follows: ‘UniqueID’, ‘bridge’ (1 for true and 0 for false), ‘tunnel’ (1 for true and 0 for false).

**Figure 2:**
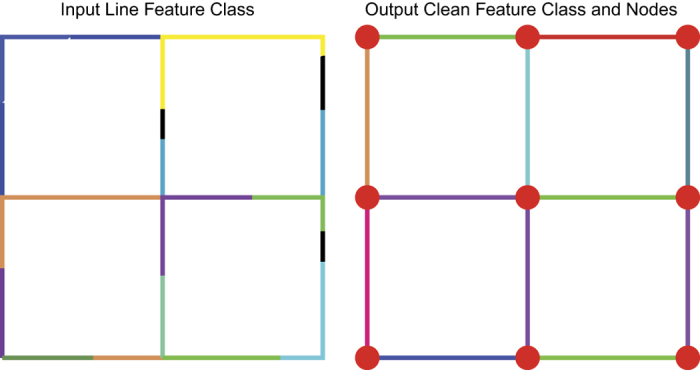
Cleaning up the OSM input data followed by creating the edges and nodes feature classes.

Our method solves two major topological problems with OSM line data. First it produces nodes and edges, only at locations where road intersections exist, which result in a non-planar graph file whereas if edges do not spatially intersect their line intersections are not considered as nodes (Fig. 1). This finding is significant because most line features consist of multiple segments between the intersections of two lines (i.e., many roads are artificially split in multiple segments). Most network analysis tools produce nodes at the start and end of all of these segments; inflating the actual number of nodes and edges, and reducing the length of most road segments. As illustrated in Fig. 2, our toolset, produces no extra nodes or edges, and enables an accurate calculation of edge length using ArcGIS which automatically takes into account the curvature of the road segments (e.g., in meters).

The data for the 80 most populated cities in the world are based on world atlas website (Table 1), and were collected from the OSM database during first two weeks of June 2015 (For a map of all the cities in the dataset see: https://goo.gl/DB576i). Table 1 lists the data sources used to create the data set. To build the road networks of the cities, we used boundary shapefiles to clip the road network from OSM data sets. We projected our data set using WGS 1984 UTM projected coordinate system (See http://geokov.com/education/utm.aspx) which enables our tool to calculate edge length in meters.

### Code availability

The dataset of 80 road networks from the most populated cities in the world was accessed from OpenStreetMaps and processed with our tool created in ArcGIS 10.2 Model Builder with an advanced (ArcInfo) license. The planar version of the tool was created with the same software package. Both tools, with a thorough tutorial on how they can be used, have been made publically available as an ArcGIS toolbox in CSUN’s website (http://csun.uic.edu/data.html#GISF2E) and on Figshare (Data Citation 1). With the required ArcGIS licenses, both tools can be used to create new datasets. Moreover, the tools can be freely downloaded, modified and improved to fit future research needs.

## Data Records

In order to create the final datasets (Data Citation 2), we created an ArcGIS tool (Data Citation 1) and utilized it to create a dataset of 80 road network shapefiles and edge lists. Essentially, our tool creates two new GIS layers, one with all nodes and one with all edges as well as an edge list in a Comma-Separated Values (CSV) file. An edge list is a list of all edges/links in the network with start node ID, end node ID, and edge ID. These unique ids correspond to the points and lines in the generated GIS files, and can be later converted back to any GIS platform to conduct analysis or spatial visualization. More specifically, an edge list is a standard method of graph representation and can be read by many graph analysis software packages or libraries (e.g., Gephi, NodeXL, or python’s igraph).

**Figure 3:**
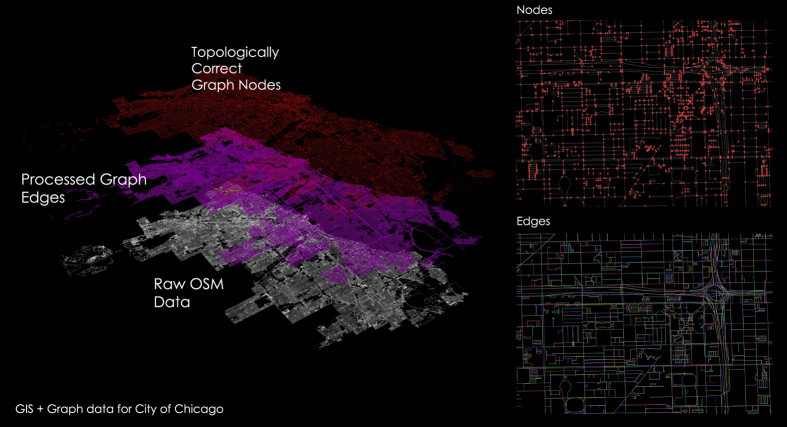
Schematic outline of the entire process to achieve nodes, edges and edge lists for a sample city like Chicago.

The datasets are released in the Figshare account, handled by the Complex and Sustainable Urban Networks (CSUN) Laboratory at the University of Illinois at Chicago. For each city, the data consist of two shapefiles, one for nodes and one for edges (Fig. 3), and one edge list (e.g., Table 2) for each network (e.g., Boston_Nodes.shp; Boston_Links.shp; Boston _Edgelist.csv).

## Technical Validation

The original roads data sets, as well as the tunnel and bridge information, along with the positional accuracy of the data, are validated by the OSM community (see http://wiki.openstreetmap.org/wiki/Accuracy). The resulting crowdsourced datasets have varying levels of positional and geographic accuracy depending on the location of the data^25^. In many cases the quality of the data has been constantly improving^26^. The data generated with our tool is first cleaned, noting that all duplicate nodes and edges are handled within the tool, then the data integrity and cleanness checked by running it through the topology validation tool provided in ArcGIS. The topology tool checks for overlapping edges, nodes, or edges that are not connected to a node. Finally, the edge lists were tested by conducting simple graph analyses and by joining the data back to GIS shapefiles. In other words, the data is accurate if all of the nodes and edges present in the CSV file generated correspond to actual nodes and edges present in the two GIS layers.

## Usage Notes

The main objective of this article is to present a protocol to convert any line feature data in GIS into a workable network format, consisting of a list of edges, a node layer, and an edge layer. This protocol was created as an ArcGIS tool and applied to the 80 most populated urban areas in the world using OSM data (where overlapping lines were taken into account). Another tool was made available for planar analyses where no edges intersect, or additional intersection data is not available.

Overall enormous amounts of GIS data sets are becoming increasingly available. Simultaneously, the study of spatial systems as graphs and networks has emerged as a substantial field in the research community. The protocol introduced along with the ArcGIS tool and the data made available for the 80 urban areas further strengthen these recent advances, and can lead to the study of more spatial systems as networks, from rivers and water pipelines, to telecommunication and electrical networks.

The GIS protocol consists of three major steps:

1. Inputting lines, cleaning up the data and creating nodes Feature Class.
2. Generating edge list data.
3. Cleaning up the results, and generating the CSV edge list output.

There is a comprehensive tutorial for using this GIS tool available at http://csun.uic.edu/data.html#GISF2E (Accessed 19 Jan 2016) and on Figshare that explains all the process in detail (Data Citation 1). The tools are available as an ArcGIS Toolbox which allows users and researchers to modify the toolbox to fit their data needs.

## Additional Information

**How to cite this article:** Karduni, A. *et al.* A protocol to convert spatial polyline data to network formats and applications to world urban road networks. *Sci. Data* 3:160046 doi: 10.1038/sdata.2016.46 (2016).

## Supplementary Material



## Figures and Tables

**Table 1:** Sources of raw data acquisition.

**Data**	**Websites where data were collected**	**Accessed Date**
Cities information	http://www.worldatlas.com/citypops.htm	1–14 Jun 2015
Road shapefiles	http://download.geofabrik.de/	1–14 Jun 2015
Boundaries	http://www.gadm.org/country	1-14 Jun 2015

**Table 2:** Sample edge list for small part of Boston’s road network.

**XCoord**	**YCoord**	**START_NODE**	**END_NODE**	**EDGE**	**LENGTH**
329880.2	4594299	1	2	1	14.64321
329880.2	4594299	1	3	2	24.43203
329880.2	4594299	1	6	3	272.6202
329874.6	4594286	2	1	1	14.64321
329904.6	4594298	3	1	2	24.43203
329793	4594549	6	1	3	272.6202
329793	4594549	6	7	5	82.32773
